# Preliminary development of a bystander intervention scale for depression and the examination of socio-demographic correlates amongst Singapore university students

**DOI:** 10.1186/s40359-021-00573-y

**Published:** 2021-04-30

**Authors:** Wei Jie Ong, Jue Hua Lau, Edimansyah Abdin, Shazana Shahwan, Janrius Chong Min Goh, Gregory Tee Hng Tan, Ellaisha Samari, Kian Woon Kwok, Mythily Subramaniam, Siow Ann Chong

**Affiliations:** 1 Research Division, Institute of Mental Health, Buangkok Green Medical Park, 10 Buangkok View, Singapore, 539747 Singapore; 2 School of Social Science, Nanyang Technological University, Singapore, Singapore

**Keywords:** Bystander intervention, Depression, Students, Scale, University, Singapore

## Abstract

**Background:**

Despite peer involvement having a positive impact on help-seeking behavior, there is a lack of a scale quantifying the possibility of an individual intervening upon noticing peers who show signs of depression. The aims of this study were to (1) develop a bystander intervention scale for depression that assesses the likelihood of university students intervening when a peer shows signs of depression based on the theory of bystander intervention, (2) identify the underlying factors contributing to the behavior, and (3) explore the socio-demographic correlates of the scale.

**Methods:**

The proposed scale, the Bystander Intervention Scale for Depression (BISD), is a 17-item self-reported questionnaire that was developed based on existing bystander intervention theory and inputs from mental health experts. Data was collected as part of a larger study to evaluate the effectiveness of an anti-stigma intervention amongst university students from a local university. A total of 392 participants were recruited. Exploratory factor analyses were performed to identify the underlying factor structure. Multiple linear regressions were conducted to explore the socio-demographic correlates of the scale.

**Result:**

Four key factors were identified for the proposed scale: (1) *Awareness of depression among peers*; (2) *Vigilance towards possible symptoms of depression*; (3) *Knowledge on how to intervene*; (4) *Acceptance of responsibility to intervene*. Having experience in the mental health field was associated with all factors while having family members or friends with mental illness was associated with all factors except for *knowledge on how to intervene.* Students of older age were associated with higher *vigilance towards possible symptoms of depression* and *knowledge on how to intervene.* Those of non-Chinese ethnicity were associated with *acceptance of responsibility to intervene*.

**Conclusion:**

This study provides a preliminary tool to assess bystander intervention in depression amongst university students. This study identifies sub-groups of the student population that require more education to intervene with depressed peers and also informs the development of future strategies.

## Background

Depression is well-established as one of the most common psychiatric illnesses, with a global prevalence rate of 4.4% [1]. A systematic review of depression prevalence in university students identified 24 independent studies and reported that the mean prevalence rate of depression amongst the population was 30.6%, ranging from 10% to as high as 85% across multiple countries between 1990 and 2010 [2]. Moreover, depression is widely recognized as a debilitating illness that can negatively impact students’ physical health, cognitive functioning, social skills, and academic performance [3–6]. However, a large proportion of people with depression remain undiagnosed, fail to seek treatment, or experience a delay in seeking professional help [7, 8]. Based on the data from the World Mental Health Surveys initiative conducted across 15 countries, the percentage of those with a mood disorder who sought treatment within a year of onset ranges between 6.0 and 52.1% [8]. Moreover, among those who sought treatment, there was a median delay of 1.0–14.0 years before they sought professional help [8]. More importantly, a retrospective study in the United States found that only 35.7% of the university students who were newly identified with depression through a university health center’s screening initiative had sought treatment within 30 days [9].

Nevertheless, research has identified facilitators to help-seeking behavior amongst individuals with depression [10, 11]. These include having positive help-seeking experiences in the past, confidentially and trust with providers, positive attitudes towards help-seeking and in particular social support and encouragement towards help-seeking [11]. A longitudinal study in Finland also found that concerns from peers were associated with recent engagement in professional help services for depression amongst youths [10]. Hence, evidence suggests that peer involvement, such as providing support and expressing concern, may act as a bridge between youths with depression and help-seeking services [10, 11]. Hence, given the positive impact of peer involvement on help-seeking behaviors, it may be beneficial to explore the likelihood of university students intervening with peers experiencing depression and understanding the latent factors contributing to this behavior. However, currently, there is a lack of a scale that can quantify the likelihood of an individual intervening upon noticing that their peers need help.

Bystander intervention refers to the act of intervening as a bystander when an individual witnesses a situation that he or she deems in need of urgent assistance. In recent years, the concept of bystander intervention is often employed in the field of interpersonal violence, bullying, and sexual abuse to guide research and inform prevention-based intervention programs [12, 13]. Most research and intervention programs were targeted at the student population where these situations often occur [14–16]. Furthermore, the relevance of the concept was underscored by the fact that peers are often present during victimization [17]. Notably, researchers have utilized Latané and Darley’s [18] bystander intervention model or its components as a theoretical framework to assess the process of bystander intervention amongst students [17, 19]. According to them, 5 sequential steps lead to bystander intervention: (1) Noticing the event; (2) Interpreting the event as an emergency; (3) Accepting the responsibility to help; (4) Knowing how to help; (5) Implementing the intervention [18]. Hence, given that university students are often surrounded by peers in schools, it may be useful to develop a scale based on the bystander intervention model to assess bystander intervention in the context of helping peers with depression.

Singapore is a multi-ethnic country with a total population of 4.03 million residents. The population comprises 74.4% Chinese, 13.4% Malays, 9.0% Indians, and 3.2% other ethnicities (Singapore Department of Statistics, 2019). About 106,000 students were estimated to be enrolled in publicly funded autonomous universities (excluding private universities) in 2018 [21]. Although the prevalence of depression amongst students in local universities is unclear, the second Singapore Mental Health Study (SMHS 2016) had reported that the lifetime prevalence rate of depression amongst Singapore residents aged 18–34, the typical age range of a university student in Singapore, was as high as 9.2% [22]. Furthermore, it is also reported that 73.0% of Singapore residents with depression failed to seek treatment during the past 12 months [23]. Hence, in order to reduce the treatment gap in Singapore, it may be worthwhile to develop intervention programs that promote peer involvement in facilitating help-seeking behaviors amongst local university students with depression. However, there is a need first to understand what predicts peer-helping behavior and the possible socio-demographic factors that influence this behavior in the local context.

Therefore, to advance research and inform and evaluate future interventions aimed at prevention, the aims of this study were to (1) develop a bystander intervention scale for depression that assesses the likelihood of university students intervening when a peer shows signs of depression based on the theory of bystander intervention, (2) identify the underlying factors contributing to the behavior, and (3) explore the socio-demographic correlates of the scale.

## Methods

### Sample

Data was collected as part of the Advancing Research Towards Eliminating Mental Illness Stigma (ARTEMIS) study. The ARTEMIS study aimed to evaluate the effectiveness of an anti-stigma intervention amongst university students from a local university. A total of 392 university students were recruited for the study. However, only 390 participants’ data were included in the analysis (2 participants were excluded for being over the age limit and incomplete participation in the study, respectively). Participants were aged between 18 and 35 years, studying in the university at the point of recruitment, and literate in English. As part of the ARTEMIS study, students participated in an anti-stigma intervention, which consisted of a talk providing information about depression and avenues for help-seeking and a sharing session by someone with a lived experience of a mental illness. Participants were assessed with identical self-administered questionnaires at 3 different time points: (1) baseline; (2) immediately after the intervention; and (3) 3 months after the intervention. However, for the current study, we have utilized only the data collected at baseline. The intervention sessions and data collection were carried out over 6 months (Oct 18–Apr 19). Written informed consent was taken from all participants before the study, and written parental consent was also obtained for participants below 21 years old. The study received ethics approval from the National Healthcare Group Domain Specific Review Board.

### Measures

#### Socio-demographics

Socio-demographic information such as age, gender, ethnicity, year of study, and discipline of study was collected with a self-administered questionnaire. Participants were also asked whether they had any past experience in the mental health field and whether they had any friends or family members with mental illness.

#### Bystander intervention scale for depression

The proposed scale, the Bystander Intervention Scale for Depression (BISD) was developed by members of the study team (i.e., MS, KK, SS, JG, WO, GT). It was developed based on the 5 steps of the bystander intervention model by Latané and Darley [18]. The study team members constructed the items by referencing other bystander intervention measures in their respective contexts. The items were created to assess the respondent’s readiness in each step of the bystander intervention in the context of depression [12, 17]. MS and SS also provided their inputs as mental health experts to further refine the items to ensure their relevance to depression. This resulted in 17 questions rated on a 5 point Likert scale ranging from 1-Strongly agree to 4-Strongly disagree.

#### Statistical analysis

Descriptive statistics were computed to describe the characteristics of the sample. Due to an insufficient sample to represent each major ethnic group in Singapore, ethnicity was categorized into 2 groups, Chinese and Non-Chinese. type of school course was also categorized into STEM (Science, Technology, Engineering, and Math) and Non-STEM (e.g. social science, humanities and business course) courses. Data were analyzed via exploratory factor analysis (EFA) in M-plus version 8.2 to identify the factors. The polychoric correlation matrix with weighted least squares with the mean and variance-adjusted chi-square (WLSMV) estimator was used. Also, oblique rotation (QUARTIMIN) was applied to obtain a more distinguished factor structure. Multiple criteria were used to determine the number of factors in the EFA: (i) visual inspection of the scree plot, (ii) eigenvalues > 1, (iii) identification of factor loadings on each factor (i.e. loadings > 0.4, without cross-loadings), and (iv) robustness of interpretability for each solution. Items were removed due to low factor loadings (< 0.4) and cross-loadings. Subsequently, Cronbach’s alphas were calculated for each factor and factor scores were generated based on sum of the relevant items. Following which, linear regression analysis was conducted to investigate the association between socio-demographic characteristics and each of the identified factors from the EFA. Statistical significance was set at p value < 0.05.

## Results

### Socio-demographic characteristic

Socio-demographic characteristics of the sample are presented in Table 1. The majority of respondents were female (60.26%) and of Chinese ethnicity (82.82%). Slightly more than half of the respondents were in a STEM course (59.50%). Furthermore, 42.56% of the respondents had a close friend or family member with mental illness and 22.22% of the respondents had past experience within the mental health field.

**Table 1:** Socio-demographic characteristic

	Mean	SD
Age	22.3	2.3
	n	%
Sex		
Male	155	39.74
Female	235	60.26
Ethnicity		
Chinese	323	82.82
Non-Chinese	67	17.18
Type of school course		
STEM courses	231	59.5
Non-STEM courses	157	40.5
Close friends or family member who has a mental illness		
Yes	166	42.56
No	224	57.44
Past experience with the mental health field		
Yes	86	22.22
No	301	77.78

*STEM* science, technology, engineering, and math

### Exploratory factor analysis

Multiple EFAs were conducted to examine the underlying factor structure for BISD. The initial examination of the scree plot and the eigenvalues for the 17-items suggested one to five factor solution. During each analysis, factor loading of the items were explored and each rotated solution was examined to identify and remove items that had low factor loading (i.e. loading < 0.4) or cross loading. This resulted in the removal of 3 items (i.e. one item for low factor loading and 2 items for cross loading). Finally, a 4 factor solution was found to be optimal (RMSEA = 0.06, TLI = 0.96, CFI = 0.98). The 4 factors were named as follows: (1) Awareness of depression among peers (α = 0.61, 3 items); (2) Vigilance towards possible symptoms of depression (α = 0.49, 2 items); (3) Knowledge on how to intervene (α = 0.75, 4 items); and (4) Acceptance of responsibility to intervene (α = 0.74, 5 items). The correlations between the factors were significant but weak (i.e. 0.15–0.32) which support the multidimensionality of this factor structure. Items in each factor are displayed in Table 2.

**Table 2:** Factors and items of the BISD scale

Items	Loading	Cronbach’s α
*Factor 1: Awareness of depression among peers*		0.611
I am aware that there are students at my university who are experiencing depressive symptoms	0.887	
I have seen students showing signs of depression at my university	0.553	
It is evident to me that someone who is experiencing depressive symptoms needs support	0.425	
*Factor 2: Vigilance towards possible symptoms of depression*		0.491
If a peer withdraws from activities that they usually enjoy, they may just be tired (Reverse scoring)	0.496	
If someone tells me that they feel hopeless about the future, I will think that it is a phase that everyone goes through (Reverse scoring)	0.793	
*Factor 3: Knowledge on how to intervene*		0.746
I know what to say to support a student who is experiencing depression	0.425	
I know who to refer a peer who show signs of depression for help	0.971	
I know who to alert when a peer is in crisis	0.866	
I would inform the university’s faculty if a peer is showing worsening signs of depression and is reluctant to seek help	0.410	
*Factor 4: Acceptance of responsibility to intervene*		0.742
If I am aware that a peer is showing signs of depression, I feel it is my responsibility to help the person	0.809	
I believe that my actions can have a positive impact on a peer who is having depression	0.521	
If I saw a peer who I did not know very well showing signs of depression, I would help them	0.706	
If I notice a peer with signs of depression, I will ask them how things are going	0.688	
If I know a peer who shows signs of depression, I would offer to accompany him to seek help	0.555	
*Items removed*		
I know what are the signs and symptoms of depression		
I don’t think there is anything I can do to help a peer who is having depression		
I feel that other students are in a better position to help a peer who is showing signs of depression		

### Socio-demographic correlates

Linear regressions found that students who had family members or friends with mental illness and past experience in the mental health field were associated with higher scores in *awareness of depression among peers*. Students who had family members or friends with mental illness, past experience in the mental health field, and were older in age were associated with higher scores in *vigilance towards possible symptoms of depression*. Those who were older and those who had past experience in the mental health field were also associated with higher scores in *knowledge on how to intervene*. Lastly, students who had family members or friends with mental illness, past experience in the mental health field, and were of Non-Chinese ethnicity as compared to Chinese where associated with higher scores in *acceptance of responsibility to intervene*. Results of the regressions are displayed in Table 3.

**Table 3:** Association between socio-demographic characteristic and awareness on the seriousness of depression

Socio-demographic	Factor 1: Awareness of depression among peers				Factor 2: Vigilance towards possible symptoms of depression				Factor 3: Knowledge on how to intervene				Factor 4: Acceptance of responsibility to intervene			
	B	p value	95% CI		B	p value	95% CI		B	p value	95% CI		B	p value	95% CI	
			Lower	Upper			Lower	Upper			Lower	Upper			Lower	Upper
Age	− 0.015	0.312	− 0.045	0.015	0.047	0.008*	0.012	0.082	0.046	0.018*	0.008	0.084	− 0.025	0.059	− 0.051	0.001
Sex																
Male	Ref				Ref				Ref				Ref			
Female	− 0.047	0.519	− 0.192	0.097	0.152	0.075	− 0.015	0.319	− 0.150	0.108	− 0.333	0.033	− 0.100	0.113	− 0.224	0.024
Ethnicity																
Chinese	Ref				Ref				Ref				Ref			
Non-Chinese	0.142	0.102	− 0.028	0.313	− 0.089	0.376	− 0.287	0.109	0.098	0.372	− 0.118	0.315	0.183	0.014*	0.036	0.329
Type of school course																
STEM courses	Ref				Ref				Ref				Ref			
Non-STEM courses	0.125	0.073	− 0.012	0.261	− 0.115	0.152	− 0.273	0.043	− 0.021	0.811	− 0.194	0.152	0.074	0.214	− 0.043	0.191
Family members or Friends with Mental illness																
No	Ref				Ref				Ref				Ref			
Yes	0.345	< 0.001*	0.213	0.477	0.238	0.002*	0.085	0.391	− 0.038	0.654	− 0.206	0.129	0.180	0.002*	0.066	0.293
Past experience in the mental health field																
No	Ref				Ref				Ref				Ref			
Yes	0.206	0.010*	0.050	0.362	0.310	0.001*	0.130	0.490	0.380	< 0.001*	0.184	0.577	0.266	< 0.001*	0.133	0.399

*CI* confidence interval, *STEM* science, technology, engineering, and math

*p* < 0.05*

## Discussion

This paper describes the development of a bystander intervention scale for depression targeted at university students. EFA of the newly developed scale identified 4 key factors: (1) *Awareness of depression among peers*; (2) *Vigilance towards possible symptoms of depression*; (3) *Knowledge on how to intervene*; and (4) *Acceptance of responsibility to intervene*. Two of the factors were similar to the steps of the original bystander intervention model [18]. *Acceptance of responsibility to intervene* and *knowledge on how to intervene* were consistent with the third and fourth steps of the model respectively. *Acceptance of responsibility to intervene* looks at respondents’ willingness in taking up the responsibility to intervene with depressed peers, while *knowledge on how to intervene* examines respondents’ confidence in their knowledge to intervene. Also, *awareness of depression among peers* captures the elements of both the first and second steps of the original model. Specifically, items associated with this dimension assess how aware the respondents are about the prevalence of depression in the university and how concerning they perceive it to be. On the other hand, *vigilance towards possible symptoms of depression* was an entirely new dimension due to the inherent context of depression. This factor is associated with items that assess how likely the respondent is to dismiss possible depressive symptoms as trivial. A qualitative study similarly reported that that peers often overlook or dismiss depressive symptoms exhibited by individuals with depression [24]. Hence, the emergence of this dimension amongst the bystander related dimensions is in line with the aim of developing a bystander intervention scale specifically for depression.

Multiple linear regression analyses were performed to investigate the association between socio-demographic factors and each identified factors. Despite the narrow age range of our participants, our study found that older students had greater vigilance towards possible signs of depression and were more likely to know how to intervene with a depressed peer. Reavley and colleagues conducted a survey amongst tertiary students and elucidated that older students were better at recognizing depression as compared to younger students [25]. Hence, it is plausible that with better recognition of depression, older students may pick up and be alert towards possible depressive symptoms more readily than younger students. Furthermore, a study conducted amongst Asian American university students found that older students have more positive attitudes towards seeking professional help with mental health issues [26]. It is possible that because of their willingness to seek help for their mental health issue, older students were more attentive and have greater awareness towards available help-seeking avenues and coping strategies which resulted in greater knowledge on how to help others who are experiencing a mental health issue. However, more research is required to explore this possibility.

Although our study found that age is associated with both vigilance towards possible signs of depression and knowledge on how to intervene with depressed peers, there was no significant association between age and readiness to accept responsibility to help peers with depressive symptoms. This lack of association can be explained with the Attribution-Emotional Model of Stigmatization [27, 28]. The model suggests that the more people perceive an individual as having control and responsibility over an negative situation, the less sympathetic they feel toward the individual, which reduces prosocial behavior [27]. A local population-based study on causal beliefs of mental illness found that 89.1% of the participants had attributed personality issues (i.e., being a nervous person or having a weak character) as a cause of depression [29]. With such beliefs, it is likely that the students had perceived depression in their peers as a result of their own personality flaw. Hence, despite better vigilance and knowledge, the perception that peers are responsible for their depression may reduce the older students’ propensity to help their depressed peers.

In addition, the study found that students of non-Chinese ethnicities as compared to Chinese were more ready to accept the responsibility to intervene with peers showing signs of depression. This is congruent to the findings of a local study which reported that Malay and Indian ethnicities were more inclusive towards people with mental illness [30, 31]. In traditional Chinese beliefs, mental illness is seen as a retribution for their ancestors’ wrongdoing [32]. It is also believed that people with mental illness have weak character [33]. Furthermore, having mental illness is detrimental to one’s “face”, a cultural construct in the Chinese community that represents social standing and power [32]. Hence with such negative connotation towards mental illness rooted in the Chinese culture, Chinese students may be less willing to associate themselves with peers showing symptoms of depression.

Respondents who had family members or friends with mental illness, or past experience in the mental health field were more aware of depression amongst peers in the university, more vigilant towards possible symptoms of depression exhibited by peers and more willing to accept the responsibility to intervene with depressed peers. However, only respondents who had past experience in the mental health field were more likely to know how to intervene with a depressed peer. Students with family members or friends with mental illness or experience in the mental health field may be less stigmatizing in general compared to their counterpart because of their personal contact with people with mental illness [34]. In which case, these individuals may be more receptive to depression related information through various sources such as media, school talks and personal observations, and more agreeable to social contact with someone who has depression [35]. Hence, this may lead to better insight of depression in the university, increased vigilance towards signs of depression and willingness to accept the responsibility to intervene with a depressed peer. However, it is possible that students with experience in the mental health field may have undergone relevant training, courses or even public forums that equip them with the necessary knowledge, competency or skill sets that facilitates supporting a person with depression as compared to those without. Therefore, this explains why students with past experience in the mental health field were more likely to know how to help someone with depression.

There are several limitations to this study. Firstly, the study is a preliminary attempt to develop a depression specific bystander intervention scale; therefore, reliability and validity have not yet been established. Secondly, EFA only identified 2 items that were associated with the dimension *vigilance towards possible symptoms of depression* which is less than the general recommended items per factor (i.e. 3–6 items) [36]. Furthermore, having low number of items associated with the dimension violates the assumption of tau-equivalence which may have contributed to the low Cronbach’s alpha [37]. Nevertheless, we decided to keep the two items because the factor loading of the items were still above the cutoff (i.e. loading > 0.4) and we believe the dimension, *vigilance towards possible symptoms of depression,* formed by the two items was important to the overall construct of bystander intervention amongst students with depression. Thirdly, confirmatory factor analysis (CFA) was not performed to reaffirm the factor structure. Although we initially planned to split the data into two for analysis, one set for EFA and the other for CFA, due to the minimum sample size of EFA and CFA (i.e. 500–1400), this was not feasible [38]. Lastly, due to convenience sampling, we did not recruit enough participants to represent each of the main local ethnicities, hence we were only able to compare between Chinese and non-Chinese. Therefore, with these limitations, there is a need for future studies to confirm the generalizability of our factor structure by conducting a CFA on another sample. We also acknowledge that there may be a need to add more items, especially in the *vigilance towards possible symptoms of depression* dimension, and further refine them, and assess the reliability and validity of the scale. Also, future studies can further explore bystander intervention in depression and its component across the different ethnicities in Singapore.

## Conclusion

Our study presents a preliminary tool to assess bystander intervention in depression amongst university students. This scale could be used to explore trends of bystander intervention in depression amongst students across different universities and evaluate the effectiveness of interventions that promote such behaviors. Four factors emerged from the scale which include *Awareness of depression among peers*, *Vigilance towards possible symptoms of depression*, *Knowledge on how to intervene*, and *Acceptance of responsibility to intervene*. The identification of these factors can inform policy makers on the areas of focus when developing future school-based, peer-involved preventive interventions. Our findings also highlight socio-demographics correlates of local university students associated with the underlying factors of bystander interventions for depressed peers. This can help to identify sub-groups of the population that require more education in intervening with depressed peers and also inform development of future strategies. Specifically, younger students need to improve their vigilance towards signs and symptoms of depression and students with minimal exposure to the mental health field require improvement in all the 4 factors identified for bystander intervention for depression. However, more research should be conducted to further explore the underlying reasons that contribute to the associations between socio-demographics and factors of bystander intervention for depression.

## Data Availability

The data set used and/or analyzed during the current study are available from the senior author MS at mythily imh.com.sg on reasonable request.

## Abbreviations

ARTEMIS: Advancing research to eliminating mental illness stigma
BISD: Bystander intervention scale for depression
CI: Confidence interval
CFA: Confirmatory factor analysis
EFA: Exploratory factor analysis
STEM: Science, technology, engineering, and math
WLSMV: Weighted least squares with the mean and variance-adjusted chi square
